# SIRT2 promotes murine melanoma progression through natural killer cell inhibition

**DOI:** 10.1038/s41598-021-92445-z

**Published:** 2021-06-21

**Authors:** Manchao Zhang, Scarlett Acklin, John Gillenwater, Wuying Du, Mousumi Patra, Hao Yu, Bo Xu, Jianhua Yu, Fen Xia

**Affiliations:** 1 Department of Radiation Oncology, University of Arkansas for Medical Sciences, Little Rock, AR 72205 USA; 2 Department of Hematology and Hematopoietic Cell Transplantation, City of Hope National Medical Center and Beckman Research Institute, Duarte, CA USA

**Keywords:** Cancer microenvironment, Oncogenes, Tumour immunology, Melanoma

## Abstract

SIRT2, an NAD^+^-dependent histone deacetylase, has been shown to play a pivotal role in various physiological processes, however, its role in cancer is currently controversial. In recent years, SIRT2 has been described as both a tumor suppressor and oncogene with divergent expression and function in various malignancies. Using murine allograft melanoma models, our results suggest increased systemic expression of SIRT2 promotes tumor progression. In this study, SIRT2-overexpressing mice exhibited enhanced tumor growth and larger tumor volumes compared to their wild-type littermates. Mechanistically, systemic overexpression of SIRT2 reduces the number of tumor-infiltrating natural killer (NK) cells and suppresses NK cell function and proliferation within the tumor microenvironment (TME). Furthermore, despite the enhancing effect of NK cell depletion on tumor volume and growth rate in wild-type littermate mice, this effect was diminished in SIRT2-overexpressing mice. Lastly, pharmacological inhibition of SIRT2 increases NK cell tumor infiltration and suppresses allograft melanoma tumor growth. The findings of this study identify a dynamic functional interaction between systemic SIRT2 and NK cell activity, which controls melanoma tumor progression. Given the recent renewed interest in NK-cell-mediated immunotherapy response, SIRT2 could present a new opportunity to mediate immunotherapy response and resistance.

## Introduction

Described roles of SIRT2, a member of the sirtuin family of NAD^+^-dependent deacetylases, have been extensive and diverse, but also conflicting. SIRT2 has been implicated in a myriad of physiological processes including metabolism^1^, inflammation^2^, aging^3^, DNA repair^4^, and cell cycle checkpoints^5^. It has also been shown to play a role in genomic stability and is integral to reducing tumor occurrence in liver and breast^6–9^. However, conflicting evidence characterizes SIRT2 as an integral component of GBM and melanoma proliferation and tumorigenicity^10,11^. With this study, we sought to further explore the complex relationship between SIRT2 and tumor progression.

SIRT2’s established roles in inflammation and glucose and iron metabolism, all known regulators of tumor progression^7,12–15^, indicate that systemic and microenvironment SIRT2, rather than just expression in tumor cells, might have implications on progression of tumors once they have already undergone tumorigenesis. The tumor microenvironment (TME), with effects on angiogenesis, immune response, and fibroblast growth factor, is an established major determinant of long-term tumor progression^16–18^. One such example is PTEN, a well-known tumor suppressor, that inhibits tumor cell growth in tumor cell, also suppresses breast tumor progression through stromal fibroblasts^19,20^. Moreover, the importance of the tumor immune microenvironment cannot be overstated, especially in the era of tumor immunotherapy. While much attention is focused on cytotoxic T lymphocytes, many other leukocytes play a role, particularly natural killer (NK) cells, which have increasingly been characterized as tumor suppressive mediators^7,17,21–24^. Interestingly, there is evidence supporting a connection between SIRT2 expression and NK cell function within the TME^14^. In murine hepatocellular carcinoma models, the microenvironments consistently promoted SIRT2 expression in NK cells, and exogenous SIRT2 was successful in upregulating production of tumoricidal mediators^7^. SIRT2 has also been shown to increase Nrf2, an inducer of NK-cell-mediated tumor surveillance^25,26^.

It is well understood that SIRT2 is involved in maintaining genomic stability in healthy cells^27^, but there is lacking evidence for its definitive role in cancer initiation, tumor progression, and the TME. In this study, we examined SIRT2’s effect on allograft melanoma tumor progression using SIRT2 transgenic and wild-type mouse models, as well as underlying changes in NK cell function within the TME. We further demonstrated mice overexpressing SIRT2 show enhanced tumor progression as well as decreased NK cell infiltration and functional activity within the tumor. Altogether, our data demonstrate a novel role for systemic SIRT2 expression on NK cell function and tumor progression.

## Results

### Elevated systemic SIRT2 expression enhances melanoma progression in mice

**Figure 1:**
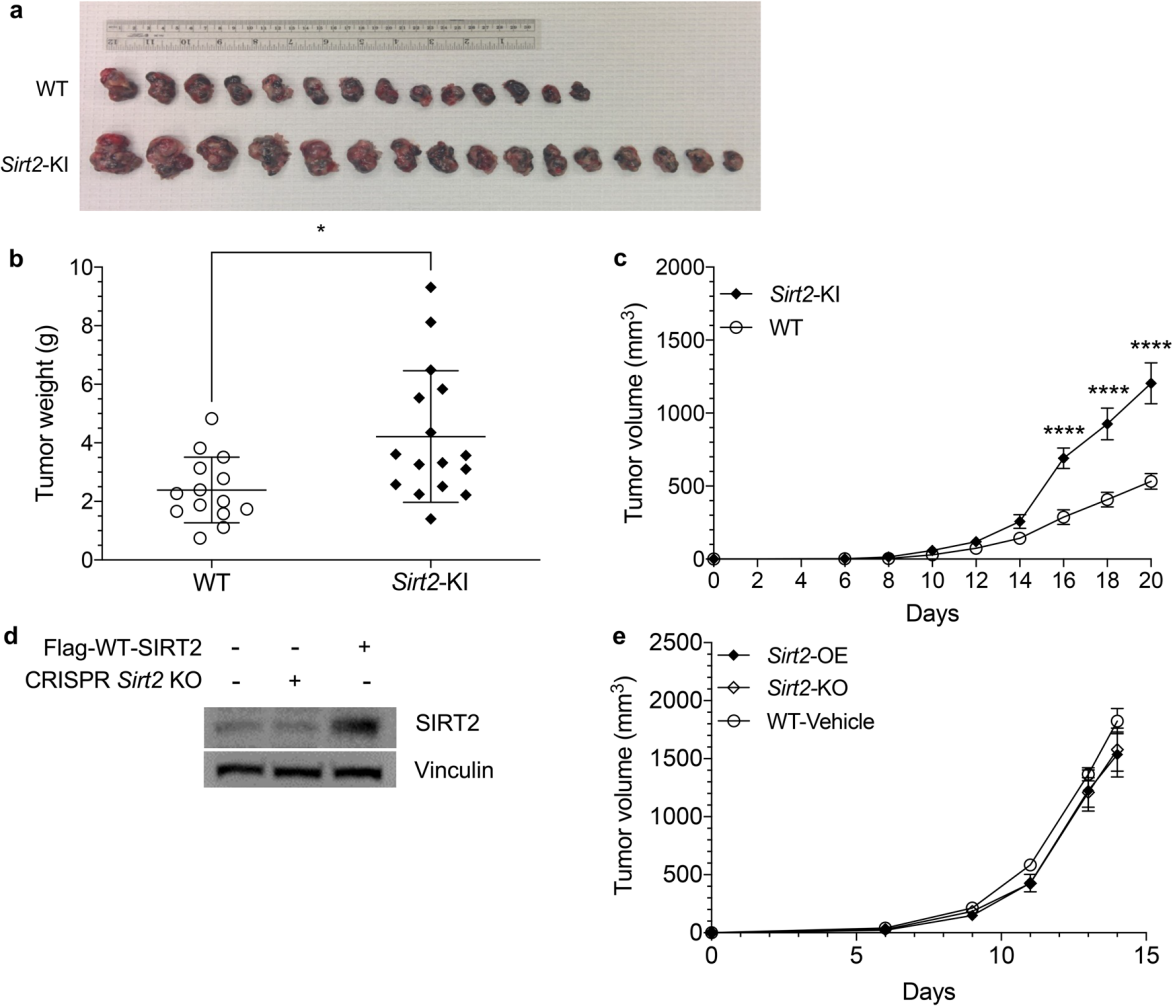
Systemic SIRT2 overexpression increases growth of melanoma tumors in mice. (**a**) Gross specimens of subcutaneous melanoma tumors harvested from *Sirt2*-KI and WT mice inoculated with 1 × 10^5^ B16-F10 melanoma cells. (**b**) Tumors in WT (n = 14) and *Sirt2-*KI (n = 16) mice were harvested on Day 20 and weighed. Student’s t test. (**c**) Tumor progression and size were monitored 20 days post injection in WT and *Sirt2*-KI mice (n = 3). Two-way ANOVA with Bonferroni correction. (**d**) Western blot showing decreased SIRT2 expression in *Sirt2*-KO B16-F10 cells following *Sirt2* knockout via CRISPR gene editing. *Sirt2*-OE B16-F10 cells overexpress SIRT2 as demonstrated by western blot following transfection with Flag-WT-*Sirt2.* (**e**) C57BL/6 WT mice were injected with WT, *Sirt2-*OE, and *Sirt2-*KO B16-F10 melanoma cells and tumor development and growth were monitored for 14 days. No significant difference in tumor progression was observed between the cell groups. n = 10. Two-way ANOVA with post-hoc Bonferroni correction. Data points are mean values ± SEM.*P < 0.05 and ****P < 0.0001.

The roles of intracellular SIRT2 as either a tumor suppressor or oncogene have been reported in several cancers including liver, breast, brain, and skin melanoma. To determine whether systemic SIRT2 expression plays a role, we examined allograft melanoma tumor progression in genetic murine models in which SIRT2 was expressed normally in wild-type (WT) mice and overexpressed in transgenic *Sirt2*-knockin (*Sirt2-*KI) mice that contain 3 copies of the *Sirt2* gene^28^. Mice were subcutaneously inoculated with B16-F10 melanoma cells at the connection between the hind limbs and the abdomen, and the tumor growth rate was closely monitored for 20 days before harvesting and measuring the weight and volume of the tumors (Fig. 1a). Both WT and *Sirt2*-KI mice had tumor formation rates of 100%; however, *Sirt2*-KI mice developed tumors that were growing significantly faster than those harvested from WT mice. The tumors from *Sirt2*-KI mice weighed more (Fig. 1b, *P* = 0.0103) and consistently progressed to a larger volume past 14 days after inoculation (Fig. 1c,* P*  < 0.0001). Utilizing E0771 mouse breast cancer cells, we repeated the experiments in *Sirt2*-KI and WT mice and observed them for 27 days. As in the melanoma model, *Sirt2-*KI mice exhibited faster tumor progression in terms of tumor volume (Supplemental Fig. S1B*, P* = 0.0045, 0.0055, 0.0013, and 0.0001) and final weight (Supplemental Fig. S1C,* P* = 0.0003).

Because previous studies have reported intracellular SIRT2 plays a role as a tumor suppressor due to its impact on genomic stability, we used the CRISPR/Cas9 lentivirus and Flag-tagged transfection systems to manipulate SIRT2 expression in B16-F10 melanoma cells to determine whether intracellular SIRT2 status is responsible for the observed tumor growth. B16-F10 cells with vector control (Vehicle), *Sirt2* knockout (*Sirt2-*KO), and *Sirt2* overexpression (*Sirt2-*OE*)* (Fig. 1d) were inoculated subcutaneously into wild-type mice using the subcutaneous melanoma model. We observed no difference in their tumor formation, growth rate, and tumor volume over 15 days (Fig. 1e), which suggests that intrinsic tumor cell SIRT2 expression is not important to control tumor progression, despite its role in genomic stability and cancer initiation^6,7,9^. We therefore began looking outside of tumor cells to determine what controls their progression. With this, we conclude that systemic, rather than intra-tumor, SIRT2 expression positively correlates with murine melanoma tumor progression.

### Increased systemic SIRT2 expression is associated with inhibition of NK cell tumor infiltration and activity within the tumor microenvironment

**Figure 2:**
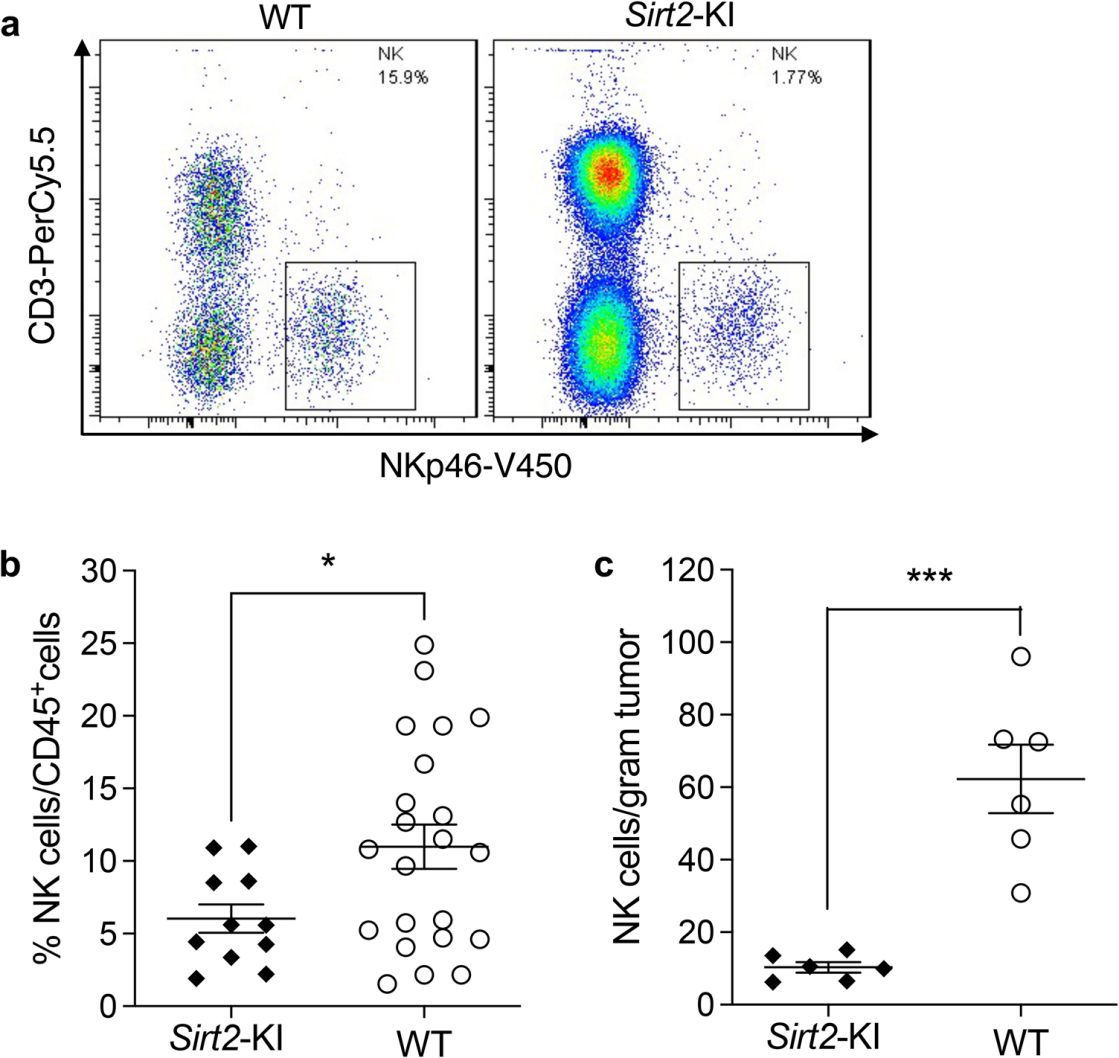
Natural killer cell distribution is altered by SIRT2 expression. (**a**) Subcutaneous melanoma tumors from WT and *Sirt2*-KI mice were harvested and analyzed by flow cytometry for the prevalence of NK cells among leukocytes. The gating strategy was designed to isolate NK cells by selecting for cells that are NKp46 positive and CD3E negative. (**b**) The percentage of tumor-infiltrating leukocytes that expressed the NK cell signature were quantified in WT (n = 22) and *Sirt2*-KI mice (n = 11). (**c**) Absolute quantities of NK cells are illustrated in *Sirt2*-KI and WT mice (n = 6). Data points represent mean values with error bars representing the SEM. Student’s t test,*P < 0.05 and ***P < 0.001.

To investigate the mechanism of enhanced tumor progression observed with overexpression of systemic SIRT2, we examined whether SIRT2 expression influenced the composition of the tumor microenvironment. Tumors in WT and *Sirt2*-KI mice displayed no significant differences in histology, particularly vascular distribution and quantity of vessels supplying the tumor (data not shown). Because of the integral role the host immune response plays in tumor progression, we examined the tumor infiltration of various immune reactive cells including the T cell, B cell, and NK cell. Samples from spleen, blood, and tumor were harvested from our allograft model and NK cells were distinguished from other leukocytes via flow cytometry by selecting for NKp46 positive and CD3E negative cells^29^ (Fig. 2a). No significant findings were observed for immune cell distributions among blood and spleen samples (data not shown) between *Sirt2*-KI and WT mice. Interestingly, the number of NK cells found within melanoma tumors was significantly decreased in *Sirt2*-KI mice compared to those harvested from WT mice in terms of percentage of total leukocytes (Fig. 2b *, P* = 0.0371) and absolute numbers (Fig. 2c). Moreover, the other immune cells examined—B cells and T cells—showed no difference in tumor infiltration based on SIRT2 expression (Supplemental Fig. S2A–C). These findings suggest possible involvement of the tumor microenvironment in tumor progression control through SIRT2-mediated inhibition of NK cell infiltration into melanoma tumors.

**Figure 3:**
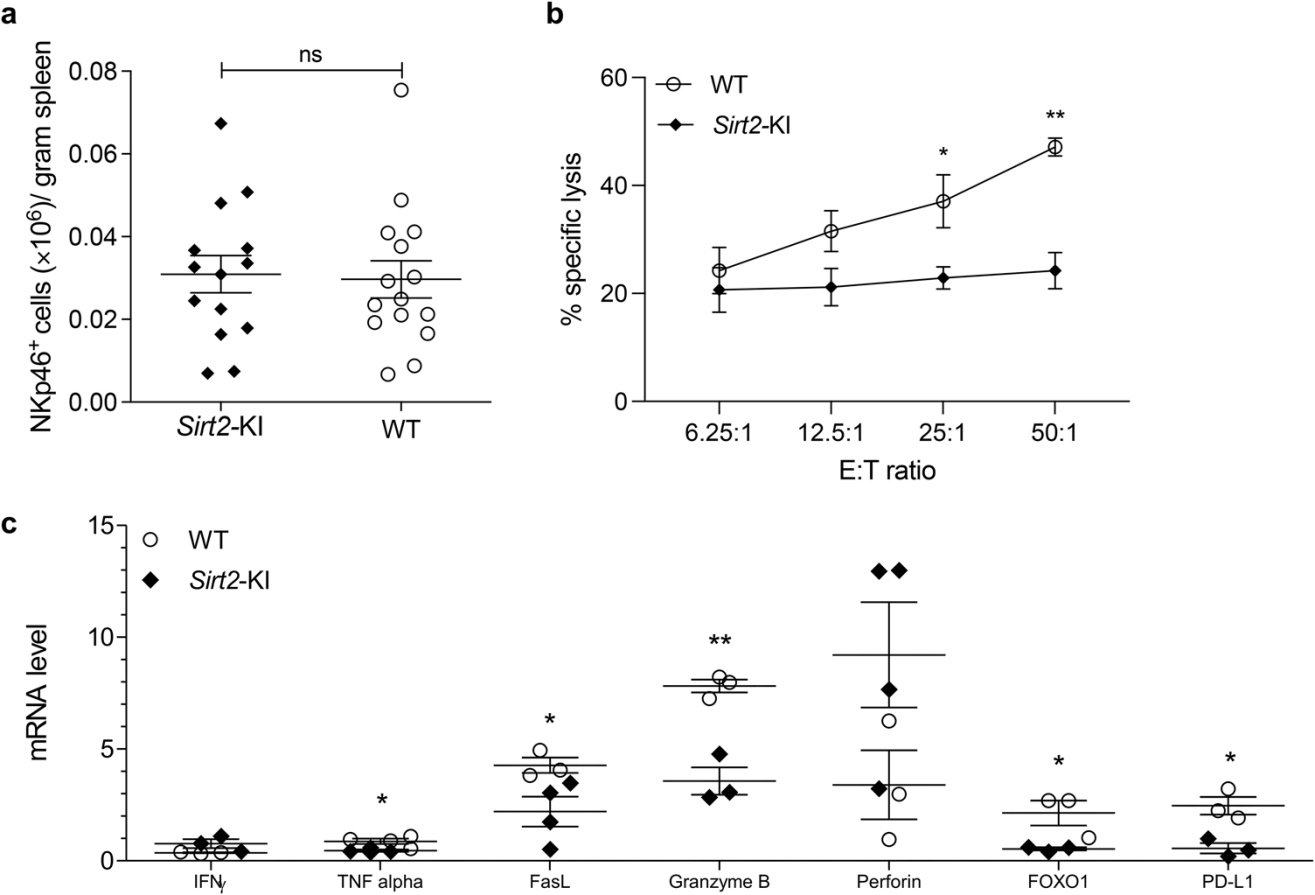
SIRT2 overexpression suppresses NK cell function ex vivo. NK cells were harvested from the spleens of *Sirt2-*KI (n = 14) and WT (n = 15) mice not bearing tumors. (**a**) NK cells were isolated and quantified by selecting for cells that are NKp46 positive and CD3E negative. Student’s t test. (**b**) Splenic NK cells were co-incubated with B16-F10 melanoma cells at E:T ratios ranging from 6.25:1 to 50:1. The cytotoxic activity of splenic NK cells is represented by % specific lysis. n = 10 and 9 for NK cells from WT and *Sirt2*-KI mice, respectively. Two-way ANOVA with Bonferroni correction. (**c**) RT-qPCR was utilized to determine the mRNA expression of cytokines related to NK cell activity. Multiple Student’s t test. n = 3 and 4. Data points are mean values ± SEM. *P < 0.05 and **P < 0.01.

SIRT2’s effect on NK cells was further characterized using NK cells harvested from the spleens of non-tumor-bearing mice so that a potential effect on NK cell function from tumors would be eliminated. There was no difference in the number of NK cells present in spleens in *Sirt2*-KI versus WT mice (Fig. 3a). To evaluate whether SIRT2 regulates NK cell effector function, we tested splenic NK cells harvested from WT and *Sirt2*-KI mice for their ability to kill B16-F10 target cells. While NK cells from both groups of mice lysed target cells, increasing the number of effector cells to target cells (E:T ratio) in two-fold increments resulted in significantly increased lysis of B16-F10 cells by NK cells from WT mice compared to that from *Sirt2*-KI (Fig. 3b, * P* = 0.0438 and 0.0015). RT-qPCR was also employed to measure cytokine mRNA expression of NK cells from mouse spleen as demonstrated in Fig. 3c. The assay revealed decreased expression of cytokine markers of activation (TNF-α^30^, PD-L1^29,31^, and FasL^32,33^), development (FOXO1^34^), and cytotoxic activity (granzyme B^35^) in *Sirt2*-KI mice, compared with those in WT mice. Because these experiments were conducted in mice not bearing tumors, we propose that systemic SIRT2 overexpression suppresses function of NK cells independent of tumor cells.

**Figure 4:**
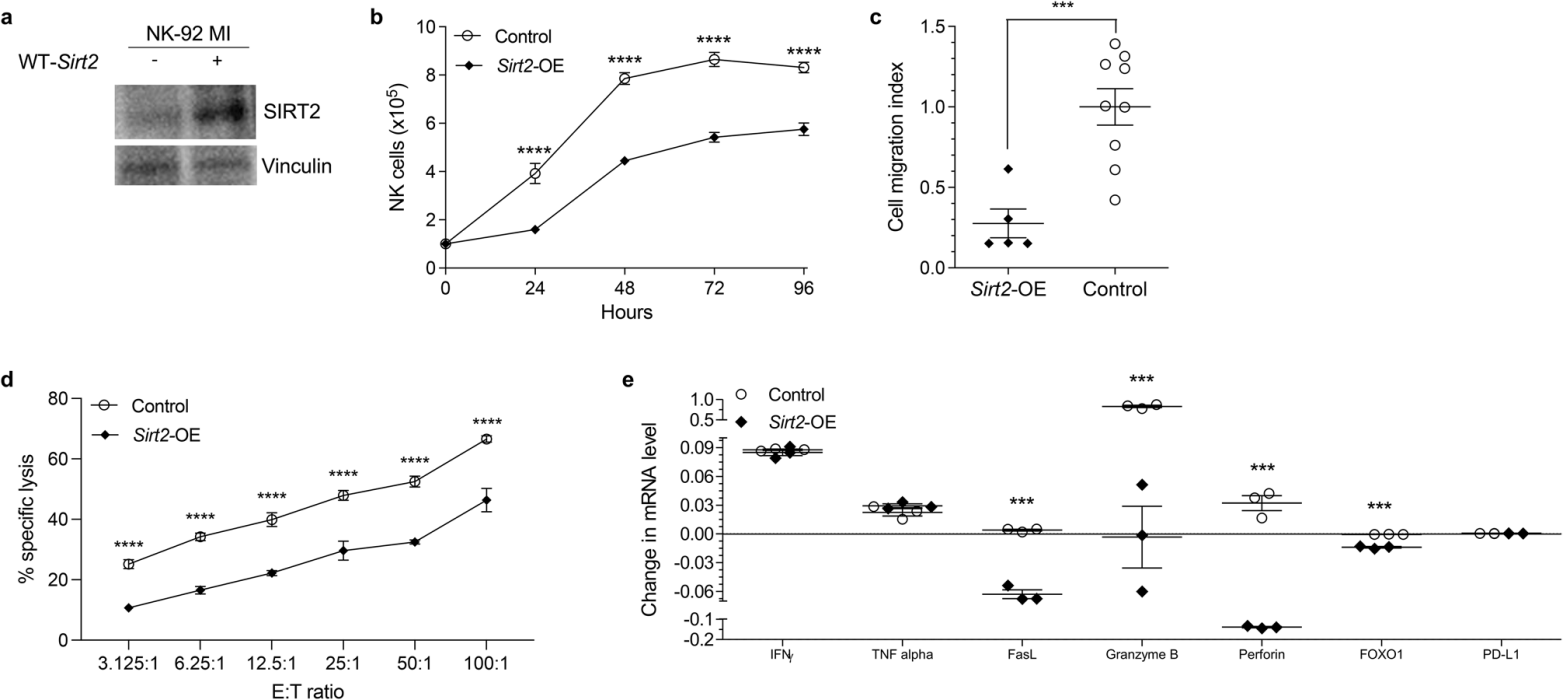
SIRT2 expression regulates human NK cell proliferation and function in vitro. (**a**) SIRT2 expression was modified through transfection and expression of exogenous *Sirt2* in human NK-92MI cells as demonstrated in the western blot. (**b**) Cell proliferation assay of NK-92MI cells with (*Sirt2*-OE (n = 4)) and without (control (n = 5)) exogenous *Sirt2*. Cell counts were performed every 24 h for a total of 96 h. Two-way ANOVA with Bonferroni correction. (**c**) Chemotaxis was evaluated using a transwell migration assay. The migration index was determined by the number of transmigrated cells after 4 h. (n = 3). (**d**) NK-92MI cells were co-incubated with K562 leukemia cells at E:T ratios ranging from 3.125:1 to 100:1. The cytotoxic activity of NK-92MI cells is represented by % specific lysis. n = 4. Two-way ANOVA with Bonferroni correction. (**e**) RT-qPCR was utilized to determine the expression of multiple NK cell cytokines following induction by IL-12. Multiple Student’s t test. n = 3. Data points are mean values ± SEM. ***P < 0.001 and ****P < 0.0001*.*

We further assessed the role of SIRT2 in NK cell function regulation with an in vitro model utilizing human NK cells. NK-92MI cells^36^ were transfected with WT-*Sirt2* to produce *Sirt2-*OE human NK cells (Fig. 4a). The effect of SIRT2 expression on cell proliferation was first evaluated. *Sirt2*-OE and WT NK-92MI cells were cultured and counted every 24 h for a total of 96 h. Compared to WT, *Sirt2-*OE cells exhibited increased proliferation as demonstrated by greater cell counts at each time point (Fig. 4b,* P* < 0.0001). To investigate whether SIRT2 expression affects NK cell migration, transwell migration assays were performed. We found that SIRT2 overexpression suppresses NK cell migration as shown in Fig. 4c (*P* = 0.001). Cytotoxic cell lysis assays were performed using NK-92MI effector and leukemia K562 target cells^37^ in increasing E:T ratios. *Sirt2*-OE NK cells demonstrated decreased specific cell lysis at every ratio point (Fig. 4d,* P* < 0.0001). Finally, control and *Sirt2*-OE human NK cells were stimulated by IL-12, and the induction of mRNA expression of NK-cell-associated cytokines was quantified through RT-qPCR. *Sirt2*-OE cells demonstrated reduced expression of FasL, granzyme B, perforin, and FOXO1 (Fig. 4e; *P* = 0.000143, *P* = 0.00004, *P* = 0.000037, *P* = 0.00007). These findings suggest that SIRT2 overexpression resulted in inhibition of NK cell activation and cytotoxic activity as described above. Immunohistochemistry staining of tumors from *Sirt2*-KI and WT mice was also performed to further evaluate NK cell proliferation within the tumor microenvironment. Double staining for Ki-67 and NKp46 revealed significantly decreased NK cell proliferation in the tumors of *Sirt2-*KI mice (Supplemental Fig. S3, *P* = 0.0211). Together, these results suggest that SIRT2 may directly regulate NK cell function, and they support the working model that increased systemic SIRT2 promotes tumor progression by inhibiting NK cell proliferation, migration, activation, and anti-tumor function.

### SIRT2’s effect on melanoma tumor progression is mediated through NK cells

**Figure 5:**
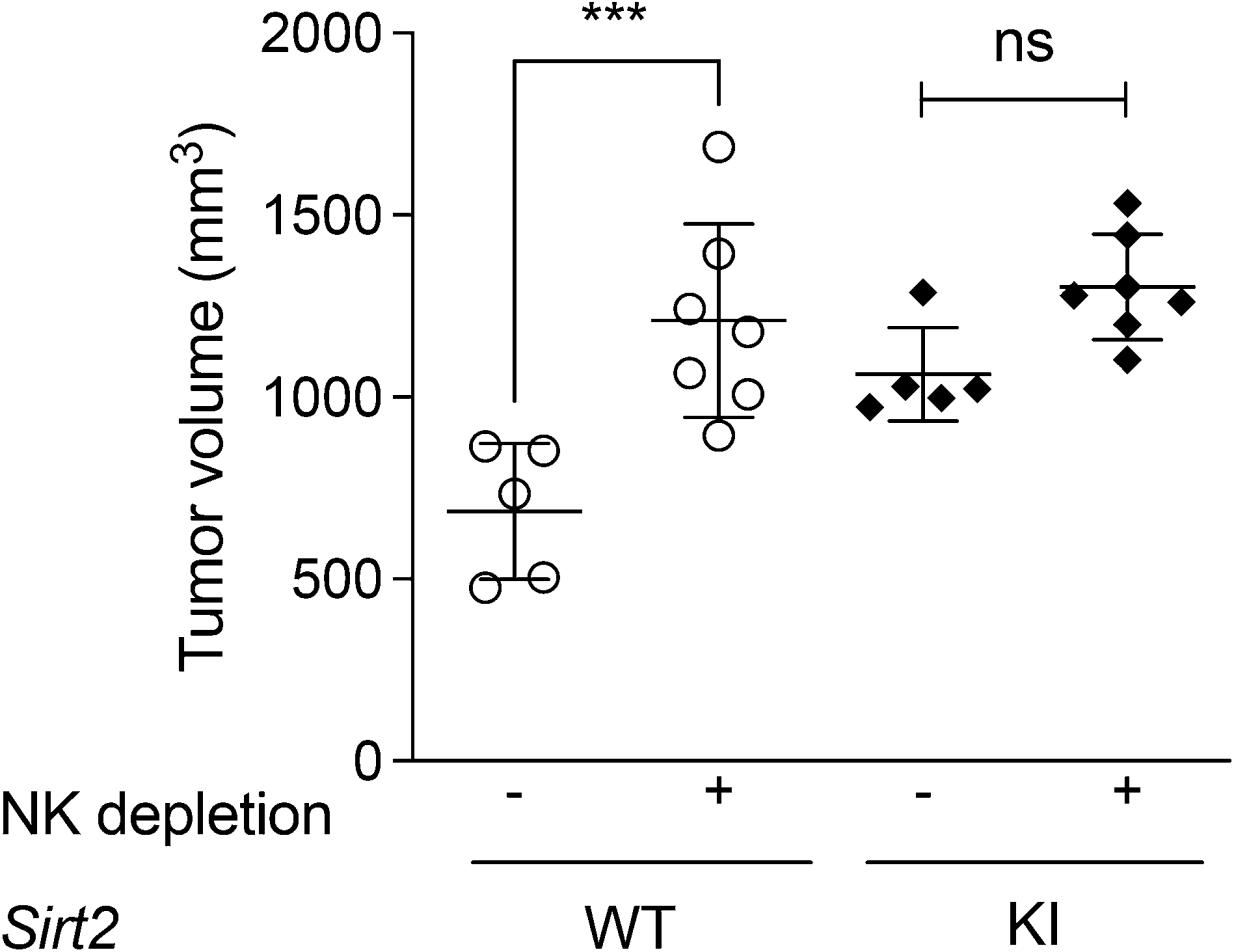
The effect of SIRT2 on tumor progression is mediated through NK cells. WT (n = 6 and 5) and *Sirt2*-KI (n = 7 and 5) mice were divided into two groups with one receiving anti-AsGM1 to deplete NK cell populations, and the other was treated with saline. Following inoculation with B16-F10 melanoma cells, tumors were monitored for growth and harvested after 21 days. Data points represent mean values ± SEM. One-way ANOVA with post-hoc Tukey HSD test, ***P < 0.001.

To examine whether the NK cell is indeed the key immune cell through which SIRT2 affects melanoma growth, we artificially inhibited NK cell function through antibody-mediated NK cell depletion to recreate suppressed NK cell in melanoma TME observed in SIRT2-overexpressing mice. Using anti-AsGM1 antibodies, we depleted the NK cell population within our mouse melanoma allograft models^38,39^. Consistent with reports from other studies^40–42^, antibody-mediated NK cell depletion in WT mice significantly promoted tumor progression compared to untreated control group, as evidenced by a 1.6-fold increase in tumor volume, and similar to that of *Sirt2-*KI mice (Fig. 5, *P* = 0.0009). On the other hand, anti-AsGM1 antibody treatment in *Sirt2-*KI mice did not significantly affect the progression of allograft melanoma tumors which have already decreased infiltrating NK cells (Fig. 2). The significant decrease in tumor progression observed in WT, but not *Sirt2*-KI, mice following NK cell inhibition further supports our hypothesis that NK cells are important in SIRT2-mediated tumor progression.

### Pharmacological inhibition of SIRT2 resumes NK cell tumor infiltration and suppresses melanoma progression

**Figure 6:**
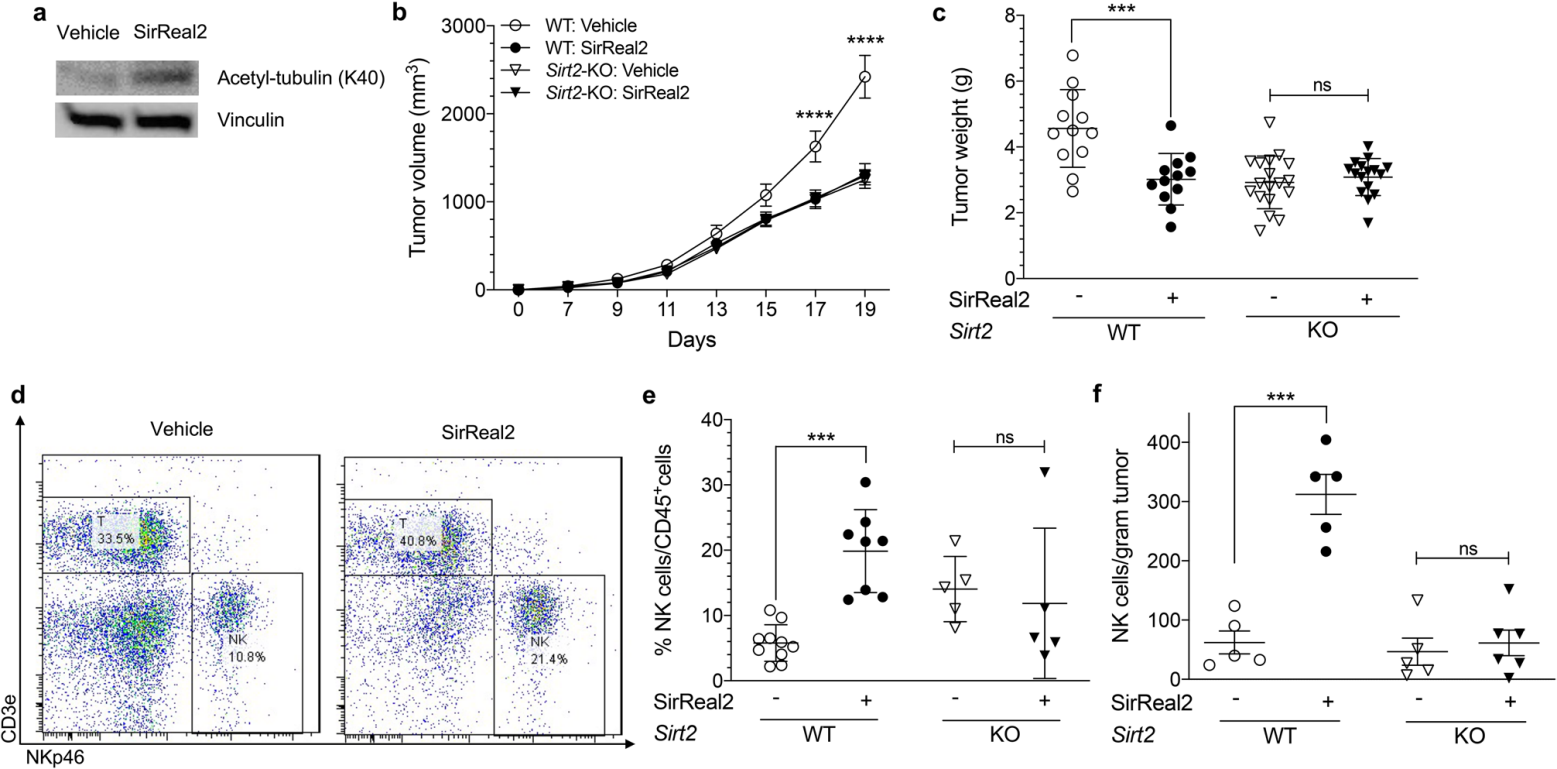
Pharmacological inhibition of SIRT2 enhances NK cell function and suppresses tumor progression. (**a**) Western blot showing decreased SIRT2 expression and deacetylase activity, as indicated by increased levels of α-tubulin K40 acetylation, in C57BL/6 mice treated with SirReal2, a small molecule SIRT2 inhibitor (n = 3). (**b**) Tumor progression and size were monitored up to 19 days following injection of B16-F10 cells into WT and *Sirt2-*KO mice treated with SirReal2 or vehicle (n = 12 and 18 for WT and *Sirt2*-KO). Two-way ANOVA with Bonferroni correction. (**c**) Tumors were harvested on Day 19 and weighed. (**d**) Subcutaneous melanoma tumors from WT mice were harvested and analyzed by flow cytometry for the prevalence of NK cells among leukocytes. The gating strategy was designed to identify NK cells as cells that are NKp46 positive and CD3E negative. (**e**) Abundance of NK cells as a percent of total leukocytes infiltrating melanoma tumors in WT (n = 10 and 8) and *Sirt2*-KO (n = 5) mice treated with vehicle or SirReal2 was quantified. (**f**) Absolute number of tumor-infiltrating NK cells in *Sirt2*-KI and WT mice. (**c**,**e**,**f**) One-way ANOVA with post-hoc Tukey HSD. Data points are mean values ± SEM. ***P < 0.001 and ****P < 0.0001.

After establishing a relationship between NK cell suppression and SIRT2-mediated melanoma growth, we hypothesized that pharmacological inhibition of SIRT2 function in vivo would have a therapeutic effect on tumor progression. SirReal2 in vivo inhibition of SIRT2 in mice was confirmed, via Western blot, by measuring the levels of acetylation of K40 (AcK40) on α-tubulin, a classic SIRT2 substrate in the liver (Fig. 6a). WT mice were treated with SirReal2 before and after inoculation with B16-F10 melanoma cells, and tumor development was monitored for 19 days. We also included *Sirt2-*KO mouse as a genetic control for validation of the specificity of SirReal2-mediated SIRT2 inhibition. SIRT2 inhibition with SirReal2 significantly slowed tumor progression in WT mice (Fig. 6b,* P*  < 0.0001 on Days 17 and 19; Fig. 6c,* P*  = 0.0002), but had no effect on melanoma tumor progression in SIRT2 deficient *Sirt2-*KO mice. To verify the mechanism of tumor suppression, we again examined the tumor infiltrating NK cells via flow cytometry analysis. NK cells were distinguished from other leukocytes by selecting for NKp46 positive and CD3E negative cells (Fig. 6d). We found that SIRT2 inhibition by SirReal2 resulted in a significant increase in the percentage of NK cells compared to the vehicle-treated group in wild-type mice. On the other hand, tumors in *Sirt2-*KO mice demonstrated no change in infiltration by NK cells regardless of SIRT2 inhibition (Fig. 6e,* P*  = 0.0006). The absolute number of NK cells in WT mice also increased with SirReal2 while it did not in *Sirt2*-KO mice (Fig. 6f,* P*  < 0.001). Together, these data illustrate a novel inhibitory role of systemic SIRT2 on NK cell function resulting in tumor progression promotion.

## Discussion

The NAD-dependent histone deacetylase known as SIRT2 shows promise as a therapeutic target in cancer treatment^43^. Its enigmatic role in carcinogenesis is gaining more attention as contradictory studies report it as being either an oncoprotein or a tumor suppressor^44^. Using genetic murine models, we demonstrate that systemic overexpression of SIRT2 promotes melanoma tumor progression through suppression of NK cell tumor infiltration and activity. Antibody-mediated depletion of NK cells increased tumor progression in WT mice, but not *Sirt2*-KI mice, further supporting the integral role of SIRT2. Importantly, pharmacologic inhibition of SIRT2 suppressed tumor progression, identifying a potential therapeutic target.

**Figure 7:**
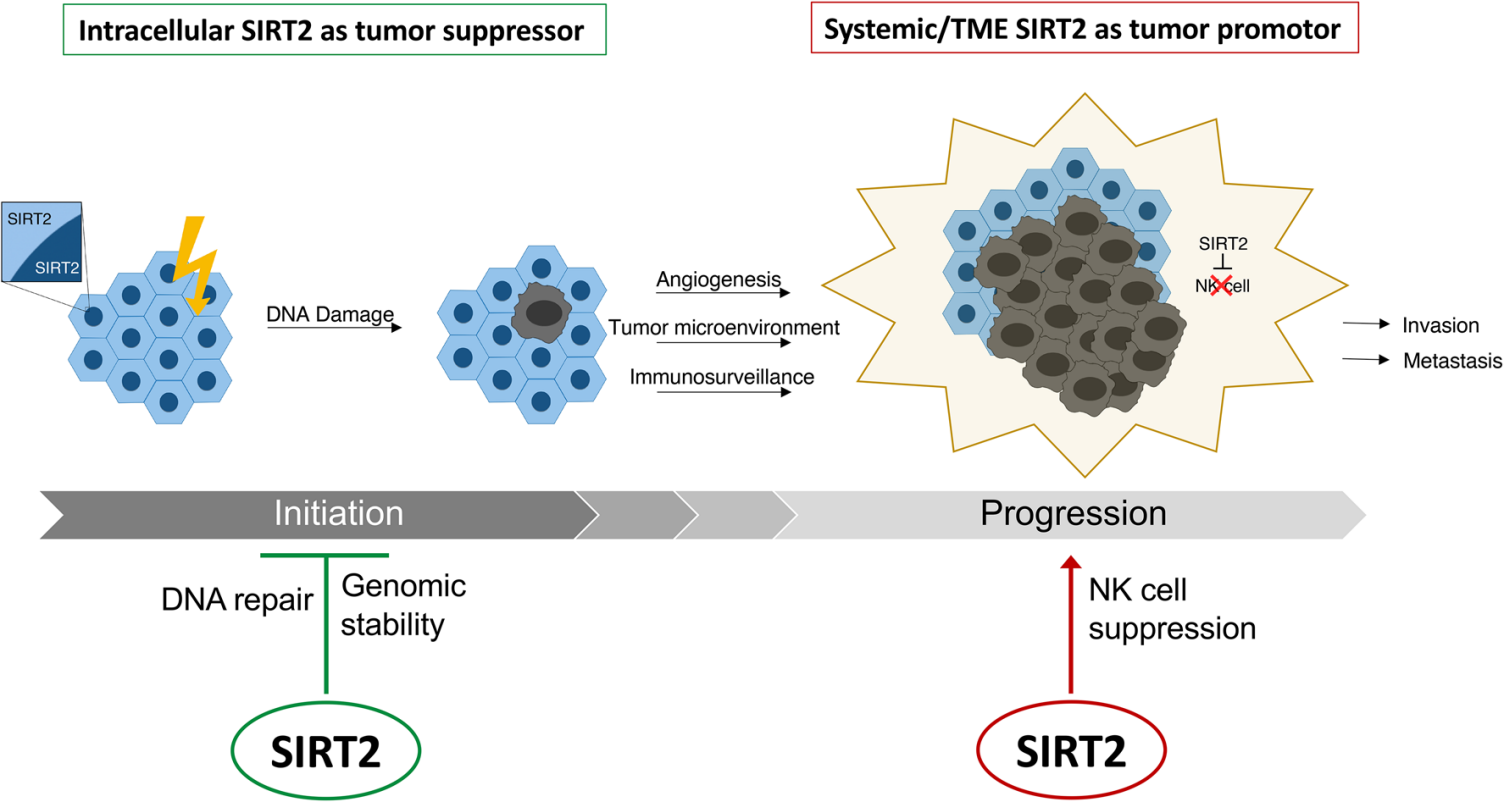
The dual role of SIRT2 in cancer initiation and progression. An illustration of SIRT2’s potential roles in cancer initiation and progression are shown with treatment strategies targeting SIRT2 proposed.

Currently, the literature supports diverging functions for SIRT2 in tumorigenesis, in a context-dependent manner, that act to modify epigenetic pathways implicated in cancer’s initiation, promotion, and progression^44–46^. Our findings illustrate the novel capacity of SIRT2 to enhance subcutaneous melanoma progression while raising the vital question of at what point does SIRT2, a tumor suppressor protein that promotes genomic integrity in healthy cells, transition into what can only be described as a tumor promoter. The data compiled from this study reveals a potential mechanism explaining systemic SIRT2’s impact upon tumor progression based on suppression of NK cell function within the tumor microenvironment. We acknowledge that our findings further add to SIRT2’s controversial role considering SIRT2 has been reported to promote the cytotoxic effects of NK cells in hepatocellular carcinoma; however, these studies differ in the types of tumors studied and the approach to SIRT2 expression manipulation. The inherent differences in hepatocellular carcinoma and melanoma could have substantial impacts on tumor progression, just as we have seen in studies looking at breast cancer and glioblastoma^8,10^. Furthermore, the effect of SIRT2 expression on NK cell tumoricidal activity was investigated using SIRT2 knockdown within liver NK cells^7^, a strategy that results in genetics vastly different from the *Sirt2*-KI mice used in our model. Combined with previously published studies, this could support a dual role for SIRT2 as illustrated in Fig. 7. Cell intrinsic SIRT2 could act as a tumor suppressor during initiation through DNA repair and genomic stability while systemic SIRT2 promoting progression of established tumors through NK cell suppression.

Our results identify NK cells as a key player in SIRT2-mediated tumor progression, suggesting that tumors generated in *Sirt2*-KI mice develop more aggressively due to a deficiency of NK cells infiltrating in TME. In addition, tumor-infiltrating NK cells also exhibit decreased functional activity and proliferation. SIRT2’s impact on NK cells within the tumor microenvironment invokes the need for additional studies examining SIRT2’s effects on other NK cell processes such as maturation, migration, and cytotoxic properties as these issues are not currently characterized. Understanding these connections is crucial as emerging data describes NK cells as a key constituent in checkpoint immunotherapy response and resistance^24^. Interestingly, NK cells were recently shown to increase the abundance of stimulatory dendritic cells within the microenvironment of human melanoma, which enhanced cytotoxic T cell function required for anti-PD-1 immunotherapy^47^. Perhaps SIRT2 expression could act as a predictor of immunotherapy response and provide a target to reverse treatment resistance and non-response through NK cells. Considering up to 80% of patients do not respond or develop resistance to immunotherapy, elucidating targets to overcome these outcomes is imperative.

In this study we strove to provide evidence that NK cell is a mediator of SIRT2’s effect in tumor progression. Multiple mechanisms could be involved in SIRT2 regulation of NK cell function. SIRT2 could also inhibit NK cells through inhibition of NK cell maturation. Despite a decrease in the number of tumor-infiltrating NK cells (Fig. 2), flow cytometry of bone marrow aspirates indicate an increase in NK cells in *Sirt2*-KI mice compared to WT mice (data not shown). This may indicate that SIRT2 affects NK cell development and maturation, an interesting question to be addressed in future study.

Current literature certainly suggests SIRT2 plays a role in immune response although direct evidence linking SIRT2 to NK cells though specific immune cells or cytokines is not yet clear. While our study provides evidence of SIRT2’s promotion of tumor progression through NK cell suppression, other immune cells, including T cells, may also be affected by SIRT2 expression. Given the functional interplay between NK cells and PD-L1, a protein widely known for T cell suppression and tumoral promotion^29^, regulation of NK cell function through PD-L1 or T cell mediation is certainly an interesting question to be explored in the future. While we acknowledge that our assay using CD3 (Supplemental Fig. S2B) is not comprehensive and does not evaluate T cell differentiation or function, the focus of this study was instead NK cells.

Macrophages can regulate NK cell function through both soluble factors and cell-to-cell interactions^48^. SIRT2 interacts with macrophages, providing a potential link between SIRT2 and NK cells. Cancer-associated fibroblasts are also capable of inducing NK cells^49^. Interestingly, SIRT2 inhibition activates fibroblasts via the MDM2 pathway^50^. Finally, SIRT2 promotes the cytotoxicity of endothelial cells under oxidative stress, and interactions between NK and endothelial cells have been identified in immune surveillance, inflammation, and wound healing^48^. Our initial screening of tumor-infiltrating leukocytes revealed a difference only in the number of NK cells while T cells, and B cells were similar between WT and *Sirt2*-KI mice (Supplemental Fig. S2). For this reason, we focused on investigating the functional interaction between SIRT2 and NK cells. We are identifying a novel relationship between SIRT2 and NK cells in the promotion of tumor progression. Meanwhile, whether other immune cells are also potential SIRT2 targets is yet to be determined.

The role of SIRT2 in the immune response beyond cancer is also an interesting question. In general, SIRT2 has been shown to suppress the immune reaction in a variety of illnesses including sepsis^51^, chronic staphylococcal infection^52^, and tuberculosis^53^. Ethanol exposure increased SIRT2 expression and reduced the inflammatory response to sepsis. SIRT2 deficiency was also shown to promote phagocytosis by macrophages leading to increased survival of mice with chronic staphylococcal infection. Lastly, SIRT2 inhibition in mice infected with *Mycobacterium tuberculosis* reduced bacillary load, decreased disease pathology, and increased protective immune responses. Many of the mechanistic studies examining SIRT2’s effect on specific immune cells focus on macrophages and demonstrate a SIRT2-dependent suppression. Specifically, SIRT2 inhibits inflammation through negative regulation of NF-kB p65 subunit^54^. Deficiency of SIRT2 was also shown to inhibit lipopolysaccharide-induced NF-kB activation as well as reactive oxygen species and nitric oxide production^55^. Although infectious models are beyond the scope of this study, the effect of SIRT2 overexpression on NK cell function in infection is an interesting question that could be explored in future studies.

In summary, this work suggests a novel relationship between systemic SIRT2 expression, NK cell behavior in the tumor microenvironment, and melanoma tumor progression. This novel role of systemic SIRT2 may be relevant to the progression of carcinomas of other organs and might help explain the conflicting data describing SIRT2’s role in tumorigenesis. With further study of the relationship between SIRT2, NK cells, and tumor progression, a new target for the challenges of immunotherapy could be developed.

## Methods

### Cell lines and culture

The B16-F10 *Mus musculus* skin melanoma and E0771 breast cancer cell lines of C57BL/6 origin were obtained from Dr. Michael Ostrowski’s laboratory at Ohio State University. K562 chronic myelogenous leukemia cell line was purchased from ATCC (Manassas, VA). All three cell lines were cultured in RPMI-1640 Medium (Gibco, USA) supplemented with 10% (v/v) fetal bovine serum (FBS) (Invitrogen, USA), 100 μg/mL penicillin, and 100 μg/mL streptomycin (Gibco, USA). Immortalized human NK cell line NK-92MI was a gift from Dr. Peter Sun (NIAID/NIH) and was grown in Minimum Essential Medium-alpha without ribonucleosides and deoxyribonucleosides but with 2 mM l-glutamine and 1.5 g/L sodium bicarbonate. To make the complete growth medium, the following components were added to the base medium to achieve a final concentration of 12.5%: 0.2 mM inositol, 0.1 mM 2-mercaptoethanol, 0.02 mM folic acid, horse serum. All cells were maintained in a humidified environment with 5% (v/v) CO_2_ at 37 °C and were in the logarithmic growth phase when harvested for injection with ~ 50% confluence in B16-F10 cell line. *Sirt2-*knockout (*Sirt2*-KO) B16-F10 cells were generated by CRISPR/Cas9 gene editing. Mouse SIRT2 CRISPR KO lentivirus vector targeting 5′-GTCATCTGTTTGGTGGGAGC-3′ was purchased from Applied Biological Materials Inc. (Richmond, BC, Canada). Third generation lentivirus was packed as previously described^56^. B16-F10 cells were infected by lentivirus and selected in the medium containing 2.0 µg/mL puromycin to obtain stable B16-F10 *Sirt2*-KO cell line. B16-F10 and NK-92MI cells were also transfected with Flag-tagged WT-*Sirt2* using FuGENE HD (Promega, Madison, WI) as before^56^ to get *Sirt2*-overexpressing (*Sirt2*-OE) stable B16-F10 and NK-92MI cell lines after selection with 1000 µg/mL neomycin and 750 µg/mL neomycin, respectively. For NK-92MI cell treatment, both NK-92MI/Vector and NK-92MI/WT-*Sirt2* cells were treated with 10 ng/mL/10^6^ cells IL-12 (R and D Systems, Minneapolis, MN) or PBS for 24 h^57^.

### Cell proliferation

10^5^ WT or *Sirt2*-KI NK-92MI cells were plated per well in multiple 48 well cell culture plates and maintained growth in a CO_2_ incubator. The number of viable cells in one plate was counted using a hemocytometer after trypan blue staining every 24 h for a total of 96 h. Cell proliferation was demonstrated as fold number of viable cells to 10^5^ cells.

### Animals

SIRT2^+/+^ (C57BL/6) mice were purchased from The Jackson Laboratory. *Sirt2* transgenic (*Sirt2*-KI or *SIRT2tg*^28^) mice and wild-type littermates with a C57BL/6 background were obtained from the Sinclair Laboratory (David A. Sinclair, PhD) in the Department of Genetics at Harvard Medical School. SIRT2^−/−^ C57BL/6 (*Sirt2*-KO) mice were obtained from Tiago F. Outeiro (Department of Neurodegeneration and Restorative Research, Center for Nanoscale Microscopy and Molecular Physiology of the Brain, University Medical Center Göttingen). All mice were bred in the facilities of the Department of Laboratory Animal Medicine (DLAM) at the University of Arkansas for Medical Sciences. All procedures were approved by the University of Arkansas for Medical Sciences Institutional Animal Care and Use Committee (IACUC). All mouse experiments were performed in accordance with NIH regulations and ARRIVE guidelines about the use and care of experimental animals. Appropriate numbers of tumor cells were suspended in ice-cold phosphate-buffered saline (PBS) and checked for viability using trypan blue staining. Only when cell viability was greater than 90% was the cell batch considered for injection.

### Mouse subcutaneous melanoma allograft model and treatment

For the subcutaneous melanoma model, 6–8-week-old *Sirt2*-KI and wild-type littermates were inoculated subcutaneously with 1 × 10^5^ B16-F10 melanoma cells resuspended in 100 μL ice-cold PBS per injection into the lower flank. The day of tumor cell inoculation was designated as day 0. Measurement of tumor size began on the 6th day and continued up to 20 days post injection. For each tumor measurement, the tumor’s longest dimension (a) and the one perpendicular to it (b) were measured using digital caliper once every 2–3 days. Tumor volume (V) was calculated according to the following formula: V = π/6 × ab^2^. The volumes calculated with this formula were closely related to the weight of the tumors isolated after sacrifice (data not shown). For tumor treatment, SIRT2 was pharmacologically inhibited with the SIRT2 specific inhibitor, SirReal2 (Cat #S7845), ordered from Selleck Chemicals (Houston, TX, USA). It was dissolved in DMSO as an 84 mg/mL stock solution and stored at -20ºC. Daily vehicle or SirReal2 treatment (50 mg/kg in PBS containing 20% PEG400 and 10% Tween-20) was injected intraperitoneally (i.p.) 5 days before B16-F10 tumor inoculation and repeated once every 3 days until the end of the experiment.

### E0771 cell mouse fat pad injection and breast cancer allograft model

E0771 breast adenocarcinoma cell line was obtained from the lab of Dr. Michael C. Ostrowski (The Ohio State University). 1 × 10^6^ E0771 cells in 100 μL ice-cold PBS were injected into the mammary fat pad of each female mouse, and tumor development was followed as described in the B16-F10 melanoma model.

### NK cell detection

Mice tumor tissues were harvested from the tumor-bearing mice on days 14–15 after tumor transplantation. After measuring the tumor weight with a small weighing boat, tumor-infiltrating lymphocytes (TIL) were isolated via Ficoll-Paque Premium (GE Healthcare, Pittsburgh, PA, USA) density gradient centrifugation after mincing in cell culture medium containing cell culture dish, grinding with a 5 mL syringe plunger on and passing through the 70 µm cell strainer. At the same time, bone marrow cells were isolated as described^58^, and red blood cells were lyzed. Next, 10^6^ TILs were pelleted, re-suspended in 50 µL blocking buffer (HBSS containing 5% normal mouse serum, 1:100 Fc blocker 24G.2 antibody, cat#553141, BD Biosciences, San Jose, CA, USA), and incubated on ice for 30 min. Subsequently, the cells were re-suspended and incubated in the antibody cocktail containing 1:100–1:400 of anti-mouse CD45 [clone 30-F11, eBioscience, San Diego, CA, USA), CD19 (1D3), CD3E (145-2C11), NKp46 (29A1.4), and Fixable Viability Dye 780 (BD Biosciences] on ice for another 30 min protected from light. Finally, the cells were washed, fixed with 4% paraformaldehyde, washed again, passed through the 70-µm cell strainer lid of flow cytometry sample tubes in HBSS with 2% FBS. The samples were wrapped with aluminum foil and stored in 4 °C refrigerator until flow cytometry analysis. For NK cell detection, the stained TIL samples were tested with LSRII flow cytometer (BD Biosciences), with live cells gated first, then CD45^+^ cells, CD19^-^ cells from CD45^+^ cells, CD3E^-^ cells from CD45^+^CD19^-^ cells, and NKp46^+^ cells from CD45^+^CD19^-^CD3E^-^ cells. Data analysis was performed via FlowJo software (Tree Star, Ashland, OR, USA). The final result is presented as either %NK cells/CD45^+^ cells or number of NK cells/gram tumor tissue.

### Depletion of NK cells in vivo

To deplete the NK cells in subcutaneous melanoma bearing mice, the C57BL/6 and C57BL/6 *Sirt2*-KI mice were subcutaneously injected with B16-F10 cells on day 0 as described above. All mice in the NK-cell depletion groups were intraperitoneally injected with 50 μL of antibodies against Asialo-GM1 (AsGM1, Wako, Osaka, Japan) combined with 50 μL of distilled water before the injection of the tumor cells and then once a week thereafter (on days-3, 4, 11, and 18). The mice in control groups were intraperitoneally injected with 100 μL distilled water. On days 14–15, mice in the NK-cell depletion groups and the control groups were euthanized for harvesting of samples. The mice were monitored daily and euthanized when moribund.

### Mouse splenic NK cell isolation and cytotoxicity assay

NKp46 cells were isolated from the spleens of *Sirt2*-WT and *Sirt2*-KI mice using the mouse anti-NKp46 microbead kit (cat# 130–095-390, Miltenyi Biotec, Auburn, CA, USA) according to the manufacturer’s instruction after grinding on the 70 µm cell strainer and red blood cell lysis. For cytotoxicity assay, calcein AM was purchased from ThermoFisher (Eugene, OR) as a 1 mg/mL solution in dry dimethyl sulfoxide. Target cells, B16-F10 or K562 cells, were resuspended in complete medium at a final concentration of 10^6^/mL and incubated with 15 μM calcein AM for 30 min at 37 °C with occasional shaking. After two washes in complete medium, cells were adjusted to 10/mL. The test was performed in U bottom 96-well microtiter plates (Nunc). Effector NK cells were distributed with six replicates at effector:target (E:T) cell ratios of 6.25:1, 12.5:1, 25:1, and 50:1 and with at least six replicate wells for spontaneous (only target cells in complete medium) and maximum release (only target cells in medium plus 2% Triton X-100). After incubation at 37 °C in 5% CO_2_ for 4 h, 75 μL of each supernatant was harvested and transferred into new plates. Samples were measured for fluorescence intensity using a BioTek microplate reader (Winooski, VT) (excitation filter: 485 ± 20 nm; emission filter: 528 ± 20 nm). Specific lysis was calculated according to the formula^59^:

$$ {\text{\% ~specific~lysis}} = \left( {\frac{{{\text{test~release}} - {\text{spontaneous~release}}}}{{{\text{maximum~release}} - {\text{spontaneous~release}}}}} \right)~ \times ~100\%  $$

### NK RT-qPCR

At least 10^6^ mouse NKp46 cells from *Sirt2*-KI and WT mice and NK-92MI cells were used to extract total RNA using TRIzol reagent (Thermo Fisher, catalog number: 15506026), and reverse transcription was performed with 1.0 µg total RNA in 20 µL reaction using High Capacity cDNA Reverse Transcription kit (Thermo Fisher, catalog number: 4368814). The TaqMan probes used are listed below.

**Table:** 

Genes detected	Mouse TaqMan assays	Human TaqMan assays
IFNγ	Mm01168134_m1	Hs00898291_m1
TNFα	Mm00443258_m1	Hs00443258_m1
Granzyme B	Mm00442837_m1	Hs00188051_m1
Perforin	Mm00812512_m1	Hs00169472_m1
FasL	Mm00438864_m1	Hs01904942_s1
Foxo1	Mm00490671_m1	Hs00231106_m1
PD-L1 (CD274)	Mm03048247_m1	Hs00204257_m1
Rplp0	Mm00725448_s1	Hs00420895_gH

Rplp0 was used as the housekeeping gene. For each PCR reaction, 1 μL cDNA was mixed with 10 μL TaqMan Fast Advanced Master Mix (Thermo Fisher, catalog number: 4444554) and 1 μL of TaqMan primers. Samples were added to 8 μL of water (for a total volume of 20 μL) in MicroAmp Fast 96-well Reaction plate (0.1 mL, ThermoFisher, catalog number: 4349607). All reactions were run in triplicate on an ABI StepOnePlus Real-Time PCR System (Applied Biosystems). Gene expression was calculated by the comparative C_T_ method.

### NK cell migration assay

Human NK-92MI cells were washed three times with serum-free RPMI 1640 medium. Cells were then counted with a hemocytometer under microscope with trypan blue staining. 1 × 10^5^ cells in 250 µL serum-free medium were loaded onto the upper chamber of one well of the 24-well Transwell chambers (Costar) with polycarbonate filters with a pore width of 5 µm. The lower chamber was pre-loaded with 500 µL RPMI 1640 medium containing 20% FBS and 20% PBS as negative control. Migrated cells in the lower chamber were counted using a hemocytometer after trypan blue staining. At least three replicates per sample were performed. Migration index was calculated as follows:

$$ {\text{Cell~Migration~Index}} = \left( {\frac{{{\text{migrated~cells~from~FBS}} - {\text{~migrated~cells~from~PBS}}}}{{{\text{total~cells}}}}} \right)~ \times ~100 $$

### Immunohistochemistry (IHC)

4 µm mouse melanoma tissue sections were deparaffinized and rehydrated before the citrate buffer antigen retrieval method was performed to unmask the antigenic epitopes. Briefly, slides were arranged in a single layer in a staining holder, submerged in a 500 mL glass beaker with 300 mL of 10 mM citrate buffer at pH 6.0, and incubated at 95–100 °C in microwave oven for 10 min. The beaker was then removed from the microwave oven to room temperature and slides were allowed to cool for 1 h. Next, slides were incubated in 3% hydrogen peroxide for 20 min to quench endogenous peroxidase activity. The sections were washed with a solution of PBS and 0.1% Triton three times. They were incubated with 10% normal goat serum in PBS + 0.1% Triton for 30 min to block nonspecific binding sites in the tissue. The tumor sections were incubated with 1:200 (in blocking buffer) rabbit anti-NCR1 (NKp46) (Abcam, Cambridge, MA, cat#ab214468), rabbit anti-Granzyme B (Abcam, cat#4059), or mouse anti-Ki67 (Cell signaling, Danvers, MA, cat#9499) antibody at 4 °C overnight in a wet box in the refrigerator. The secondary antibody used was anti-rabbit AP and anti-mouse HRP antibody (ENZO Biochem, Farmingdale, NY, USA). After staining with the HIGHDEF IHC chromogen substrate DAB and AP (ENZO Biochem), the tissue sections were dehydrated by subsequent alcohol washings using 70%, 95%, and 100% EtOH solutions for 5 min each. Total cells were counted, or slides were imaged under the microscope (original magnification 20×; Carl Zeiss, Thornwood, NY, USA). Cell counts were performed on at least three consecutive slides with three mice in each group. DAB staining was quantified using ImageJ. Briefly, a DAB image was firstly opened and its color deconvoluted by choosing "H DAB" from the pull-down window to get three new images. The one with "Colour_2" in the title is the DAB image (Colour_1 is the hematoxylin image). Next, Colour_2 image window was selected, followed by running Analyze > Set Measurements, and selecting "Mean gray value" and "Display label". Lastly, the analysis was done by running Analyze > Measure (or press Ctrl-m). A "Results" window displayed quantification in units of intensity. DAB staining Optical Density (OD) numbers^60,61^ were calculated with the following formula where max intensity = 255 for 8-bit images:

$$ {\text{OD}} = {\text{log}}\left( {\frac{{{\text{maximum~intensity}}}}{{{\text{mean~intensity}}}}} \right) $$

For Ki-67 staining, five similar areas in the randomly selected regions in the targeted tumor tissues were measured in the tumor tissues from *Sirt2*-KI and *Sirt2*-WT mouse. For vimentin staining, the same five areas in the randomly selected regions in the DAB-staining positive tissues were measured.

### Western blot

SIRT2, Flag-SIRT2, acetyl tubulin (Lys40), and vinculin were detected using western blot analyses as previously reported^56^. The antibodies used and their dilutions were: Anti-SIRT2 rabbit polyclonal antibody (Proteintech Group, Inc, Rosemont, IL, USA), 1:500; anti-Flag mouse monoclonal antibody (Sigma, St. Louis, MO), 1:1000; anti-acetyl-α-tubulin Lys40 mouse monoclonal antibody and anti-vinculin rabbit monoclonal antibody (Cell Signaling Technology, Inc), 1:1000.

### Statistical analysis

All data are represented as a mean with standard error of the mean (SEM). The results were evaluated using two-tailed Student’s t test to compare between two groups of results and one-way ANOVA followed by Tukey multiple comparison test or two-way ANOVA followed by Bonferroni correction when more than two groups were compared (GraphPad Prism, version 6.05, GraphPad Software, Inc.). *P < 0.05 was considered statistically significant.

## Supplementary Information

Supplementary Information 1.
