# Supplementary material for: SIRT2 promotes murine melanoma progression through natural killer cell inhibition
Source: Sci Rep. 2021 Jun 21;11:12988. doi: 10.1038/s41598-021-92445-z (PMC8217567; doi:10.1038/s41598-021-92445-z)
Supplement: Supplementary file 1 — Supplementary Information 1. [file 41598_2021_92445_MOESM1_ESM.pdf]

SIRT2 promotes murine melanoma progression through natural killer cell inhibition

Manchao Zhang[1,3], Scarlett Acklin[1,3], John Gillenwater[1], Wuying Du[1], Mousumi Patra[1], Hao Yu[1], Bo Xu[2], Jianhua Yu[2], and Fen Xia[1*].

[1]Department of Radiation Oncology, University of Arkansas for Medical Sciences, Little Rock, AR, USA; [2]Department of Hematology & Hematopoietic Cell Transplantation, City of Hope National Medical Center and Beckman Research Institute, Duarte, California

[3]These authors contributed equally.

Correspondence:

[*]Fen Xia, MD, PhD, Department of Radiation Oncology, University of Arkansas for Medical Sciences, Little Rock, AR, 72205, USA; Tel: 501-686-7100; Fax: 501-686-7285; E-mail: FXia@uams.edu

Supplemental Figures

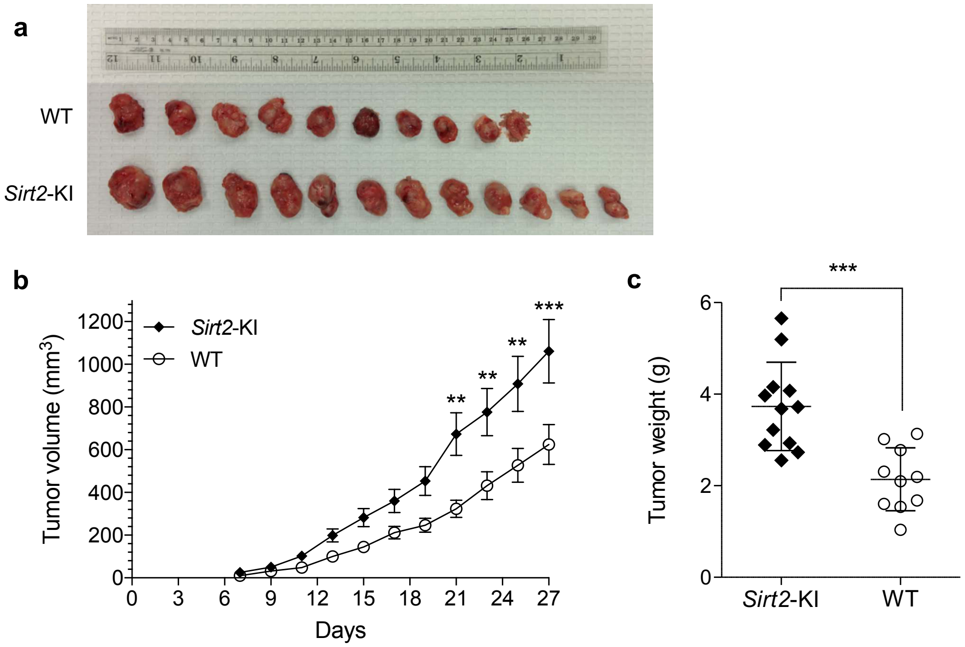

Supplemental Figure 1. Systemic SIRT2 overexpression increases growth of breast cancer tumors in mice

(A) Gross specimens of subcutaneous breast cancers harvested from *Sirt2*-KI and WT mice inoculated with 1 x10^6 E0771 breast cancer cells. (B) Tumor progression and size were monitored 27 days post injection in WT and *Sirt2*-KI mice (n = 3). Two-way ANOVA with Bonferroni correction. (C) Tumors in WT (n = 5) and *Sirt2*-KI (n = 6) mice were harvested on Day 27 and weighed. Student's t-test. Data points are mean values ± SEM. **$p<0.01$ and ***$p<0.001$.

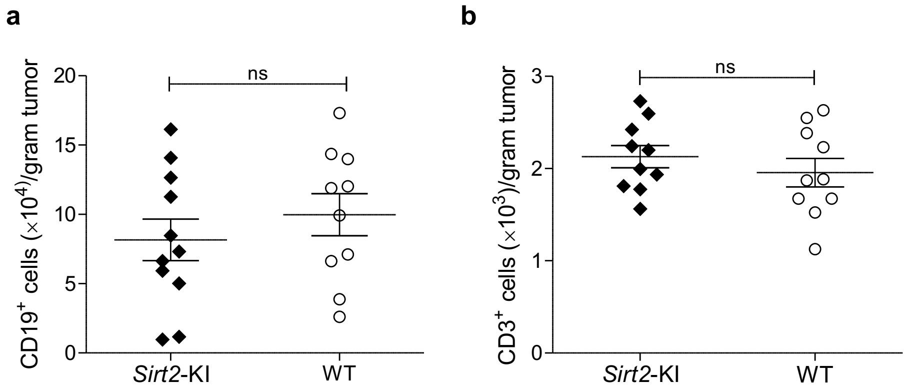

Supplemental Figure 2. SIRT2 expression does not impact tumor infiltration by B cells and T cells

B cells and T cells were isolated from the spleens of melanoma-bearing WT and *Sirt2*-KI mice and quantified using flow cytometry. (A) $CD19^{+}$ cells represent B cells. (B) CD3+ cells represent T cells. n = 3. Data points are mean values ± SEM.

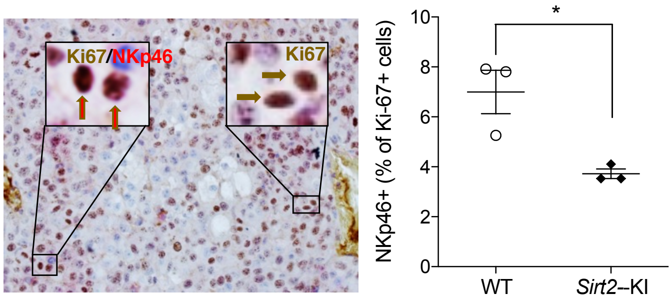

Supplemental Figure 3. NK cell proliferation is decreased in SIRT2-overexpressing mice
Immunohistochemical staining of subcutaneous melanoma samples from 3 *Sirt2*-KI and 3 WT mice was used to determine tumor-infiltrating NK cell proliferation. Images represent staining in WT mice. Cells stained positive for NKp46 were categorized as NK cells and are represented by one of three sections. Quantitative analysis is representative of 5 fields from each sample where the number of NKp46 cells divided by the total Ki-67 number to yield a percentage. Samples that expressed both the Ki-67 proliferative marker and the NK signature represent healthy NK cell proliferation. Data points represent mean values and error bars designate SEM; n = 3, Student's t-test, *$p<0.05$.

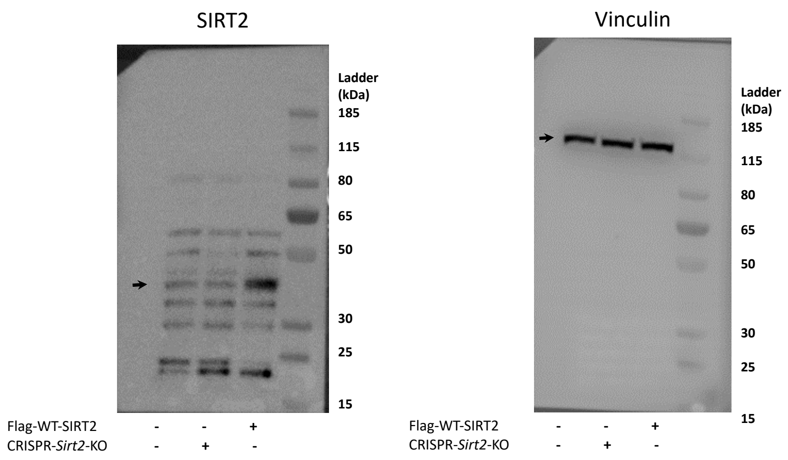

Supplemental Figure 4. Representative original complete, uncropped, unprocessed western blot corresponding to panel D of Figure 1

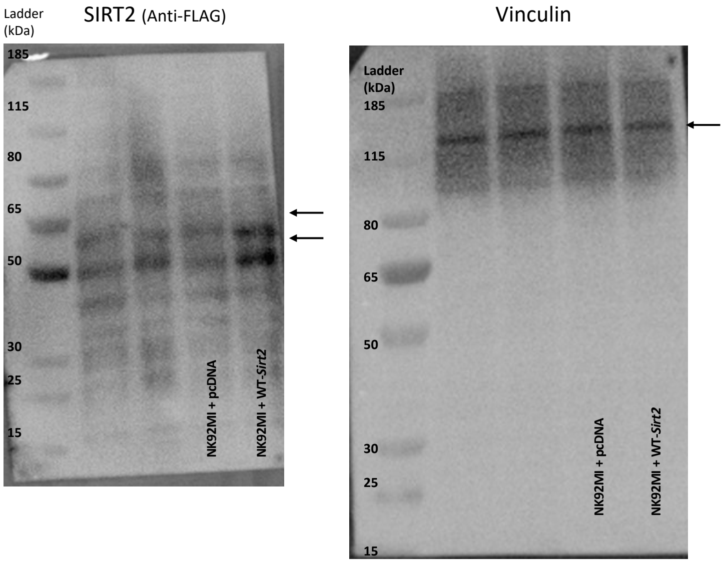

Supplemental Figure 5. Representative original complete, uncropped, unprocessed western blot corresponding to panel A of Figure 4

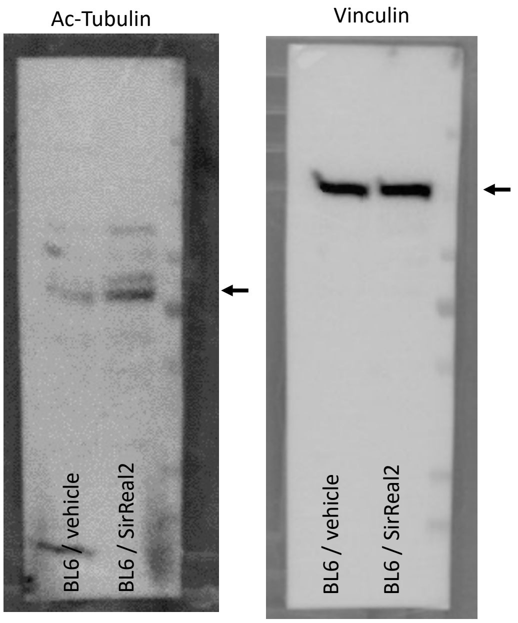

Supplemental Figure 6. Representative original complete, uncropped, unprocessed western blot corresponding to panel A of Figure 6
